# Straw mulching and nitrogen application altered ammonia oxidizers communities and improved soil quality in the alkaline purple soil of southwest China

**DOI:** 10.1186/s13568-021-01211-x

**Published:** 2021-04-07

**Authors:** Songhe Chen, Rencai Gao, Xiaoling Xiang, Hongkun Yang, Hongliang Ma, Ting Zheng, Yun Xiao, Xue Zhang, Han Li, Gaoqiong Fan, Yang Yu

**Affiliations:** 1 Key Laboratory of Crop Eco-Physiology & Farming System in Southwest China, Ministry of Agriculture, Sichuan Agricultural University, Chengdu, 611130 Sichuan Province China; 2 Soil and Fertilizer Institute, Sichuan Academy of Agricultural Sciences, Chengdu, 610066 Sichuan Province China

**Keywords:** Alkaline purple soil, Straw mulching, Nitrogen, Ammonia-oxidizing archaea, Ammonia-oxidizing bacteria, *AmoA* genes

## Abstract

Microbe-mediated ammonia oxidation is a key process in soil nitrogen cycle. However, the effect of maize straw mulching on the ammonia oxidizers in the alkaline purple soil remains largely unknown. A three-year positioning experiment was designed as follows: straw mulching measures as the main-plot treatment and three kinds of nitrogen application as the sub-plot treatment. We found the contents of soil organic carbon (SOC), total nitrogen (TN), available potassium (AK), available nitrogen (AN), available phosphorus (AP), and NH_4_^+^-N were increased after straw mulching and nitrogen application in alkaline purple soil, so did the *amoA* genes abundance of ammonia-oxidizing archaeal (AOA) and bacterial (AOB). Terminal restriction fragment length polymorphism (T-RFLP) analysis revealed that *Thaumarchaeote* (448-bp T-RF) was dominated the AOA communities, whereas *Nitrosospira* sp (111-bp T-RF) dominated the AOB communities. The community compositions of both AOA and AOB were altered by straw mulching and nitrogen application in alkaline purple soil, however, the AOB communities was more responsive than AOA communities to the straw mulching and nitrogen application. Further analysis indicated that SOC and AP were the main factors affecting the abundance and community compositions of AOA and AOB in alkaline purple soil. The present study reported that straw mulching and nitrogen strategies differently shape the soil ammonia oxidizers community structure and abundance, which should be considered when evaluating agricultural management strategies regarding their sustainability and soil quality.

**Supplementary Information:**

The online version contains supplementary material available at 10.1186/s13568-021-01211-x.

## Introduction

Nitrification is an important aspect of the soil nitrogen cycle, which helps crops to absorb soil nitrogen and regulate soil nitrogen loss (Liu et al. 2019). Ammonia oxidation is the rate-limiting step of nitrification and plays an important role in the nitrogen cycle; it is driven by ammonia oxidation archaea (AOA) and bacteria (AOB). Ammonia oxidizers contain the *amoA* gene that encodes the ammonia monooxygenase (AMO), which regulates the first step in ammonia oxidation and is usually used as the biomarker for the analysis of ammonia-oxidizer communities (Wang et al. 2019).

AOA and AOB have been found to exist widely in most agricultural soils and are influenced by soil environmental factors and tillage management practices (Wessén et al. 2010; Li et al. 2015). For example, AOA are favoured in low pH and ammonia-poor soil environments (Zhang et al. 2012), while AOB dominate nitrification in high nitrogen, and neutral or alkaline soils (Di et al. 2009). Studies have indicated that the abundance of AOA is higher than that of AOB both in alkaline and acidic purple soils (Zhou et al. 2016). However, the response of AOA and AOB community compositions and abundances for agricultural cultivation management were not consistent (Liu et al. 2015). Previous studies have shown that the application of chemical fertilization increases AOB abundance and changes its community composition, but had little influence on AOA in calcareous fluvo-aquic soil (Ai et al. 2013). Moreover, some researchers have demonstrated that organic input has no significant effect on the composition of AOA community in sandy loam (Zhang et al. 2019), while Zhalnina et al. (2012) found that the addition of organic matter increased AOA abundance and had major influence on the AOA communities in the acidic red soil. In addition, the implications of straw returning and chemical N fertilization for soil microbial community have been extensively studied. Hu et al. (2019) showed that straw returning, N fertilization, and their interactions significantly increased the abundance of AOA and AOB in paddy soil, and AOA was more abundant than AOB. Similarly, further research revealed that fertilizer combined with straw could increase AOB number, and AOA was more abundant than AOB in clay loam (Wessén et al. 2010). However, some studies indicated that adding straw did not alter the abundance of AOA and AOB significantly in sandy soil (Wu et al. 2017). Therefore, the effects of straw returning and chemical N fertilization on soil ammonia oxidizers remain inconsistent; this may be attributed to different soil environments and cultivation measures.

Purple soil is the most representative soil-type in the Sichuan basin of south-western China. This soil is formed by rapid physical weathering of sedimentary rocks of the Trias-Cretaceous system and are characterized by lithologic soil without distinct pedogenic horizons. Purple soil is classified as Orthic Entisols, according to the Chinese soil taxonomic system, and as Regosols in the FAO taxonomy or Entisols in USDA taxonomic terms (Zhou et al. 2016). Moreover, purple soil has low content of organic carbon and available phosphorus and high potential fertility (Xiao et al. 2016; Wang et al. 2018), which makes it an important agronomic soil-type. Our previous results confirmed that straw mulching could reduce water evaporation and accumulate effective rainfall to meet the growth demand of winter wheat (Yang et al. 2020). However, the effects of years of straw mulch and nitrogen application on ammonia oxidizers on the alkaline purple soil in the hilly areas are unclear. In this study, we investigated the abundance and composition of ammonia oxidizers based on straw mulching and long-term fertilization of experimental fields. The objectives of this study were to (i) elucidate the implications of straw mulching with chemical N fertilization for alkaline purple soil fertility; (ii) identify the composition and abundance of ammonia oxidizers as well as the dominated and specific ammonia oxidizers in alkaline purple soil; and (iii) evaluate the relationships among the AOA and AOB communities, as well as soil properties. We hypothesized that straw mulching and application of N fertilization could cause changes in soil properties, which may result in shifts of ammonia oxidizers abundance and community composition.

## Materials and methods

### Study sites

**Fig. 1:**
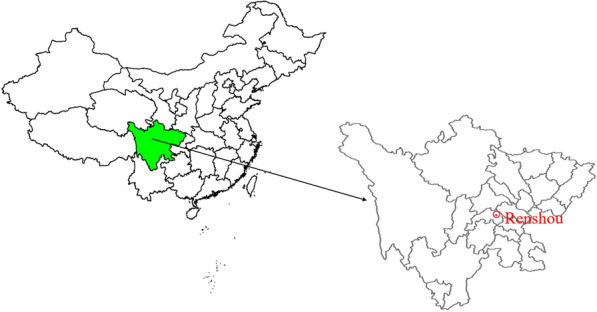
Location of the study area

The straw mulch no-tillage location test started in 2015 and was carried out in Renshou experimental base (Fig. 1, 30° 04′ N, 104° 13′ E) of Agricultural College of Sichuan Agricultural University, China. The annual average temperature of this region is about 17.4 ℃ and mean annual precipitation is 1009.4 mm. Moreover, this area is known for rain-fed agriculture, with most of the precipitation falling between April and September with a marked dry season between October and March (Du et al. 2013), with more rain for summer maize and less for winter wheat. The physical and chemical properties (0–20 cm) at the start of the experiment in 2015 were as follows: pH, 7.82 (soil: water = 1:2.5); organic carbon, 9.78 g kg^−1^; total nitrogen, 0.83 g kg^−1^; total phosphorus, 0.86 g kg^−1^; total potassium, 13.96 g kg^−1^.

### Sampling

The experiment was laid out in a split plot design, with straw mulch as the main factor and nitrogen level as the sub-plot. Each treatment was replicated three times and the plot size was 30 m^2^ (6 m × 5 m). We selected maize straw mulching at rates of 6000 (S1) kg ha^−1^ for experimental plot and 0 (S0) kg ha^−1^ for control plot, and N fertilization at rates of 0 (N0), 120 (N1), and 180 (N2) kg N ha^−1^ under combined P and K fertilization, at 75 kg P_2_O_5_ ha^−1^ and 75 kg K_2_O ha^−1^, respectively. Wheat harvest tillage horizon (0–20 cm) from plots were collected by S-shaped 5-point sampling method for one soil sample using a stainless-steel soil sampler with a diameter of 20 cm on 1st May, 2018. The sampling tool was a soil auger which had been rinsed with alcohol and deionized water. Then the roots and stones (> 2 mm) were removed from the samples. Every plot was carefully mixed to form a composite sample and transported to the laboratory in a constant-temperature box containing ice. One part of each soil sample was stored at – 80 ℃ until DNA extraction, and others were dried under natural conditions.

### Soil properties

All chemical analyses were based on air-dried soil. The pH was determined on a 1:2.5 soil/water mixture. The SOC was determined using potassium dichromate oxidation external heating method; soil TN and available nitrogen (AN) were determined using the alkaline hydrolysis diffusion method and semi-micro Kjeldahl method, respectively (Lu 1999). Soil available phosphorus (AP) was extracted using sodium bicarbonate and measured using the molybdenum blue method (Lu 1999). Soil available potassium (AK) was extracted using ammonium acetate and determined with flame photometry (P7 Double Beam UV–Visible Spectrophotometer; MAPADA Inc. Shanghai, China) (Lu 1999). Soil extractable ammonium (NH_4_^+^) and nitrate (NO_3_^−^) were extracted with 2 mol L^−1^ KCl and determined colorimetrically (Singh et al. 2010).

### DNA extraction and quantitative real-time PCR amplification

Total DNA was extracted from 0.5 g of soil using Fast DNA Spin Kit for Soil (MP Inc. CA, USA), following the manufacturer’s instructions and stored at – 20 ℃. The *amoA* gene primers of AOA were Arch-amoAF (5′-STAATGGTCTGGCTTAGACG-3′) and Arch-amoAR (5′-GCGGCCATCCATCT-GTATGT-3′) (Francis et al. 2005), and the *amoA* gene primers of AOB were *amoA*-1F (5′-GGGGTTTCTACTGGTGGT-3′) and *amoA*-2R (5′-CCCCTCKGSAAAGCCTTCT-TC-3′) (Cytryn et al. 2012). The qPCR was performed with ABI7500 Fast Real-Time PCR System (Applied Biosystems Inc. USA). Each reaction containing 12.5 µL of the SYBR Green Master Mix (Applied Biosystems, USA), 0.5 µL (10 µM) of each primer, 1 µL (20 ng/μL) DNA template and 10.5 µL of sterile water were used to make up a final volume of 25 μL. The procedure included an initial denaturation at 95 ℃ for 30 s, and then 40 cycles at 95 ℃ for 15 s, and 45 s at 55 ℃ (archaea)/60 ℃ (bacteria), 72 ℃ for 45 s. PCR products of AOB and AOA *amoA* gene were purified using PCR Purification Kit (Tiangen Biotech, Beijing, China), and then cloned using pClone007 Blunt Simple Vector Kit (Tsingke Biotech, Beijing, China), and the right gene inserts were chosen after sequencing and BLAST in GenBank on the NCBI’s homepage (https://blast.ncbi.nlm.nih.gov/Blast.cgi) to serve as standards and positive controls. The standard plasmids were quantified using a Nanodrop-2000 Spectrophotometer (NanoDrop Technologies, Wilmington, DE, USA).

The standard curves for *amoA* were created using a tenfold dilution series (10^8^–10^2^ copies) of the plasmids containing the targeted gene fragments from the soil. The standards and the DNA samples were analysed on the same plate. No amplification was detected in the negative control without template DNA. The qPCR was performed in duplicates and the efficiencies ranged from 94 to 110%, one sharp peak was observed in the melting curve for the *amoA* standard, and the R^2^-values of the standards were higher than 0.98. CT (Cycle Threshold) values were used to calculate the numbers of AOB and AOA *amoA* gene abundance according to Harter et al. (2014). PCR inhibitors in soil DNA extracts were examined by diluting soil crude DNA. Inhibition was not detected in any of the controls.

### Terminal restriction fragment length polymorphism (T-RFLP) analysis of the bacterial and archaeal *amoA* genes

The T-RFLP analysis of the *amoA* genes was used to determine both bacterial and archaeal ammonia-oxidizers community composition. The same primers of qPCR were used in the T-RFLP analyses, and each forward primer was labelled with 6-carboxyfluorescein (FAM). The PCR reaction was performed in a 25 µL volume containing 12.5 µL of 2 × Taq PCR Master Mix (Tiangen Biotech, Beijing, China), 0.5 µL (5 µM) of each primer, 1 µL DNA template (20 ng/µL), and 10.5 µL of ddH_2_O. The PCR protocols for both bacterial and archaeal *amoA* genes were replicated three times using the following programs: 5 min at 94℃ for initial denaturing, followed by 35 cycles of 94 ℃ for 30 s, 55 ℃ for 45 s, and 72 ℃ for 45 s with the final extension for 10 min at 72 ℃. After amplification, the triplicate PCR reactions were pooled and purified using the PCR cleanup Kit (Axygen Biosciences, Union City, CA, USA). The purification PCR products were digested with 10 units of restriction enzyme *MboI* (TaKaRa) at 37 ℃ for 6 h and then denatured at 80 ℃ for 30 min. The T-RFLP profiles were generated by capillary electrophoresis using an ABI Prism 3100 Genetic Analyser at Sangong Corporation (Shanghai, China). The T-RFLP lengths ranged from 50 to 500 bp in size were considered for the further analyses. The peak heights of terminal restriction fragments (T-RFs) with size differences ≤ 2 bp in an individual profile were combined and considered to be one fragment. The T-RFs with a relative abundance < 1% were excluded from further analysis.

### Clone, sequencing, and phylogenetic analysis of bacterial and archaeal *amoA* genes

To identify the main T-RFs of the AOB and AOA TRFLP profiles, bacterial and archaeal *amoA* genes clone libraries were constructed using the same primers as T-RFLP but without the 6-FAM label. PCR-generated *amoA* fragments from soil DNA were excised from agarose gels and purified using the DNA Purification Kit (Tiangen Biotech, Beijing, China), followed by molecular cloning with a pClone007 Blunt Simple Vector Kit (Tsingke Biotech, Beijing, China) into Trelief™5α Chemically Competent Cell (Tsingke Biotech, Beijing, China). Screening for the correct size inserts was performed by PCR with M13 forward and reverse primers (Tiangen Biotech, Beijing, China). A total of 180 positive clones (approximately 30 clones from each treatments) were randomly selected for restriction screening (restriction enzymes: *MboI*), and 49 typical clones were sequenced. The obtained sequences with more than 97% identity with each other were grouped into the same operational taxonomic unit (OTU) using MOTHUR (Schloss et al. 2009). Only one representative sequence of each OTU was compared using BLAST. Typical sequence in our T-RFLP experiment and their related sequences obtained by BLAST were chosen to construct the neighbour-joining tree using MEGA version 5.0 (Tamura et al. 2013). The virtual digests with *MboI* were carried out on the sequences retrieved from the clone libraries to allow the assignment of phylogenetic identity to individual T-RFs.

## Statistical analyses

The means and standard deviations of the soil physicochemical parameters and *amoA* gene diversities were performed with two-way analysis of variance (ANOVA). In the two-way tests, the significance level was *P* < 0.05. Redundancy analysis (RDA) was performed to analyse the relationships between soil properties and T-RFLP profiles of bacterial and archaeal *amoA* genes, using the software package CANOCO 5.0. The clone sequences were aligned using ClustalX (Thompson et al. 1997), and a neighbour-joining tree was constructed using MEGA 5.0. Bootstrap analysis was used to estimate the reliability of the phylogenetic reconstruction (1000 replicates) (Tamura et al. 2011).

## Results

### Soil physicochemical properties

**Fig. 2:**
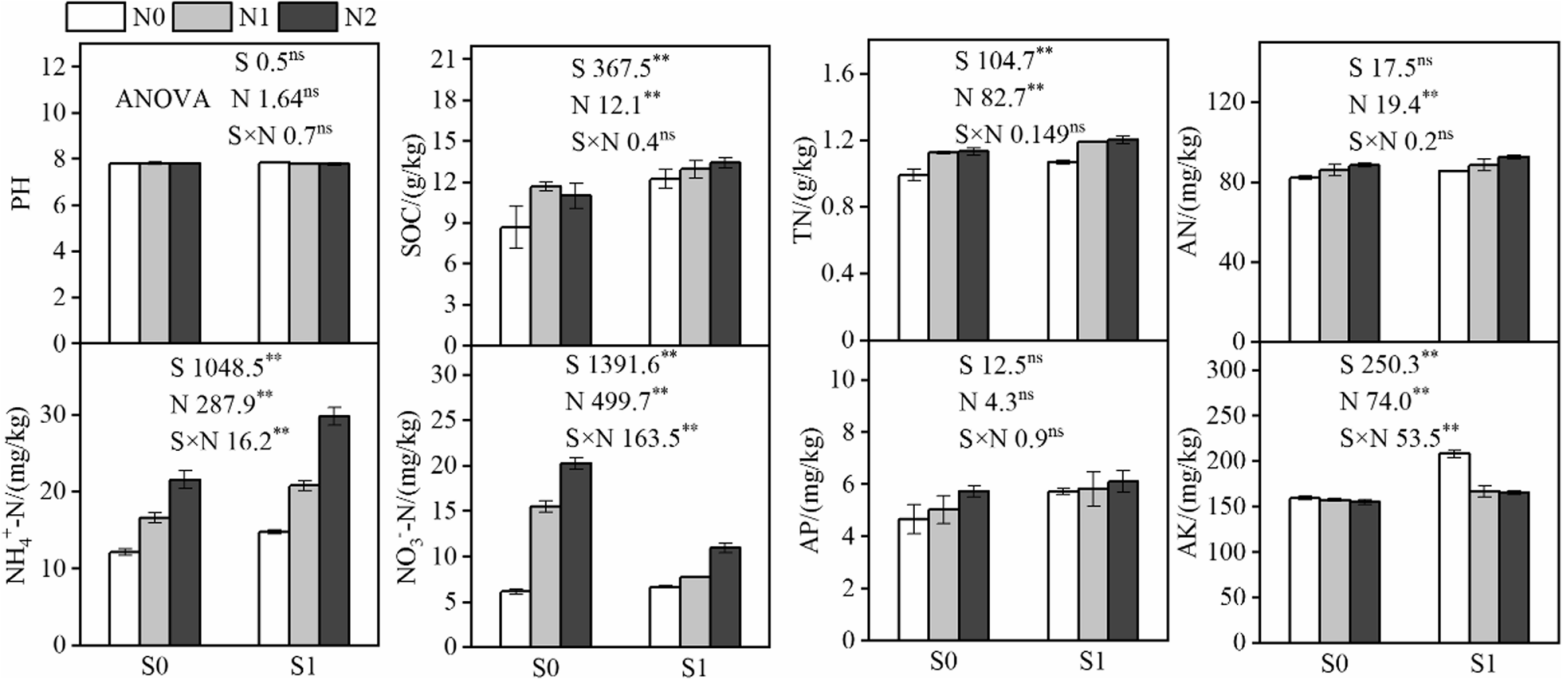
Analysis of variance of pH, SOC and TN (g kg^−1^), AN, NH_4_^+^-N, NO_3_^−^-N, AP and AK (mg kg^−1^). SOC: Soil organic carbon; TN: Total nitrogen; AN: Available nitrogen; NO_3_^−^-N: Nitrite-N; NH_4_^+^-N: Ammonium-N; AP: Available phosphorus; AK: Available potassium. S1: straw with nitrogen, S0: no straw with nitrogen, N0: no nitrogen, N1: 120 kg N ha^−1^, N2: 180 kg N ha^−1^.M, and N represent the maize straw mulch and nitrogen levels, respectively. ^*^, and ^**^ indicate significance at the 5% and 1% probability level, respectively.

**Fig. 3:**
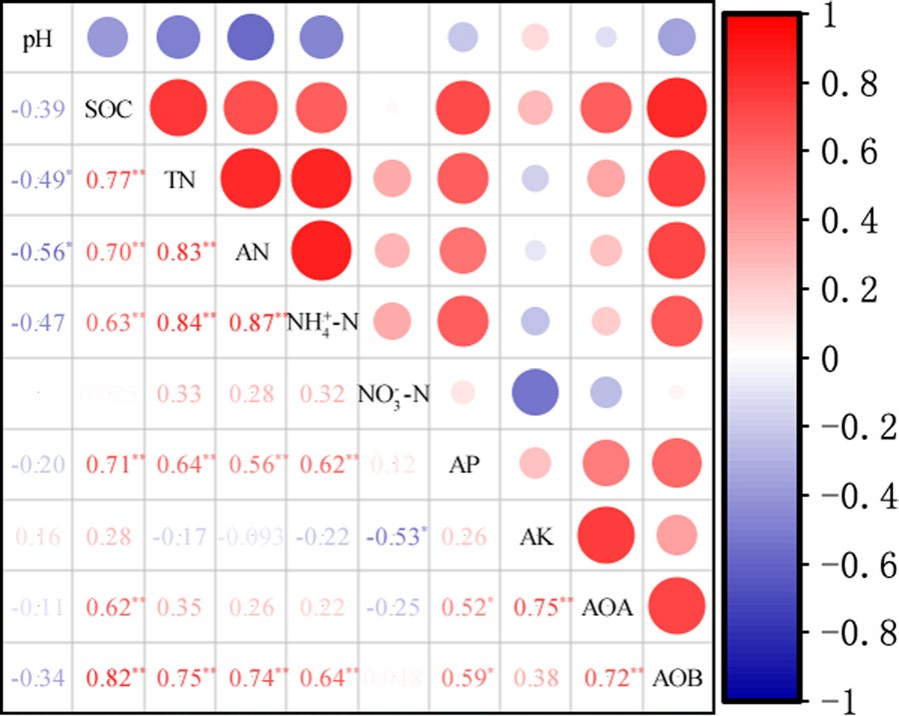
Pearson correlation analyses of soil properties and *amoA* gene abundances of ammonia oxidation archaea (AOA) and bacteria (AOB). Red represents a positive correlation, blue represents a negative correlation, and the larger the circle, the darker the colour, the stronger the correlation. SOC: Soil organic carbon; TN: Total nitrogen; AN: Available nitrogen; NO_3_^−^-N: Nitrite-N; NH_4_^+^-N: Ammonium-N; AP: Available phosphorus; AK: Available potassium. ^*^, and ^**^ indicate significance correlation at the 5% and 1% probability level, respectively

As shown in Fig. 2, soil pH was not significantly changed by straw mulching, nitrogen application, and their interaction. The soil organic carbon (SOC) content of straw mulch (S1) treated soil were 18.86 g kg^−1^, which was significantly higher than that under no straw mulching (S0) treatment of 10.46 g kg^−1^ (*P* < 0.05), and the interaction between straw mulching and nitrogen application had no significant effect on the SOC content, while SOC content was significantly increased by nitrogen application compared with no nitrogen application (*P* < 0.05). The variation trend for total nitrogen (TN), available potassium (AK), available nitrogen (AN), available phosphorus (AP), and NH_4_^+^-N were similar to that of SOC. Available potassium (AK) content was significantly increased by straw mulching, nitrogen application, and their interaction (*P* < 0.05). The available nitrogen (AN) content was significantly increased by increasing nitrogen application, with 83.93, 87.22, and 90.48 mg kg^−1^ for N0, N1, and N2 treatments, respectively. Although straw mulching with nitrogen application increased the available phosphorus (AP) content, the difference was not statistically significant. The content of NO_3_^−^-N was lower in straw mulching than that in the control groups, which had increased by nitrogen application. In addition, the correlation analysis confirmed that pH was negatively significantly correlated with TN, AN, and NH_4_^+^-N (*P* < 0.05), and there were significant or extremely significant positive relationships among SOC, TN, AN, NH_4_^+^-N, and AP (*P* < 0.01, 0.05; Fig. 3).

### Abundances of archaeal and bacterial *amoA* genes

**Fig. 4:**
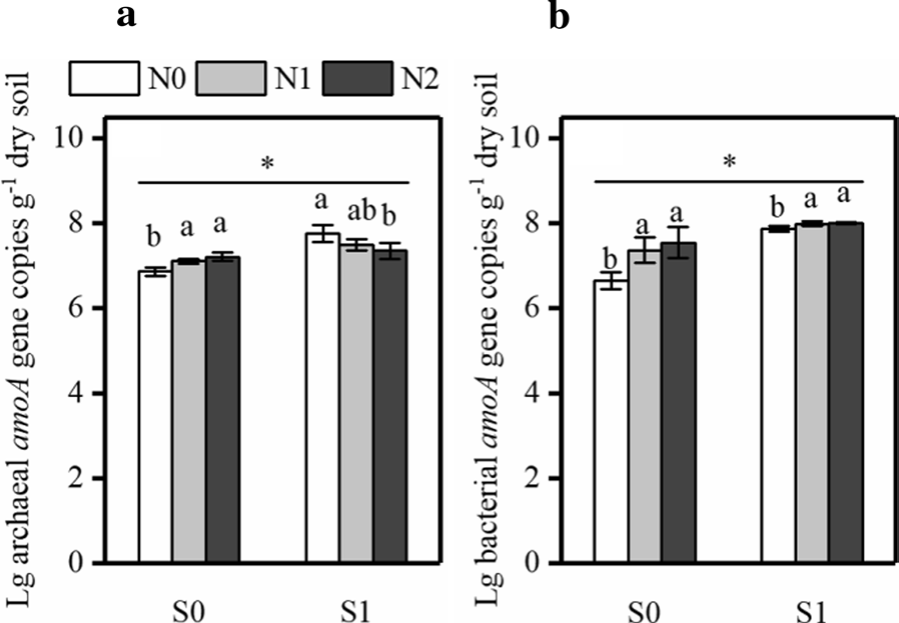
The abundance of *amoA* gene copies of ammonia oxidation archaea (**a**) and bacteria (**b**) in the different treatments. Data were mean ± S.D. S1: Straw with nitrogen, S0: no straw with nitrogen, N0: No nitrogen, N1: 120 kg N ha^−1^, N2: 180 kg N ha^−1^. ^*^, and different letters indicate significate at the 5% probability level, respectively

The abundance of archaeal *amoA* genes in soil samples significantly increased from 6.87 log 10 per gram dry soil of the control (S0) to 7.77 log 10 per gram dry soil of the straw mulching (S1) groups (*P* < 0.05, Fig. 4a). The increase of nitrogen fertilizer did not significantly influence the abundance of ammonia-oxidizing archaeal (AOA) in the sub-plot treatment. Interestingly, our results indicated that nitrogen application increased the AOA abundance in no straw mulching groups and decreased that in straw mulching groups. The combination of straw mulching and nitrogen on the significantly improved the AOA abundance (*P* < 0.05). Ammonia-oxidizing bacterial (AOB) abundance ranged from 6.64 log 10 per gram dry soil of the control (S0) to 8.01 log 10 per gram dry soil of the straw mulching (S1) treatment (*P* < 0.05, Fig. 4b), indicating that AOB abundance was significantly affected by the straw mulching, nitrogen application, and their interaction (*P* < 0.05). The ratios of AOA/AOB abundance ranged from 0.9 to 1.0 (Additional file 1: Fig. S1), indicating that the AOA abundance was lower than that of AOB and AOB was dominated in the soil. Specifically, nitrogen treatment significantly increased the *amoA* gene copies of AOB (*P* < 0.05, Fig. 4b), therefore, the AOA abundance was less sensitive to nitrogen application than AOB. Importantly, the combination of straw mulching and nitrogen application resulted in a shift in the predominant ammonia-oxidizers from AOA to AOB. Pearson correlation analyses revealed that SOC, TN, AP, and AK significantly affected the abundance of AOA *amoA* gene (*P* < 0.05), and the abundance of AOB *amoA* gene was significantly affected by SOC, TN, AN, NH_4_^+^-N and AP (*P* < 0.05, Fig. 3).

### Compositions of the ammonia-oxidizing bacterial and archaeal communities

**Fig. 5:**
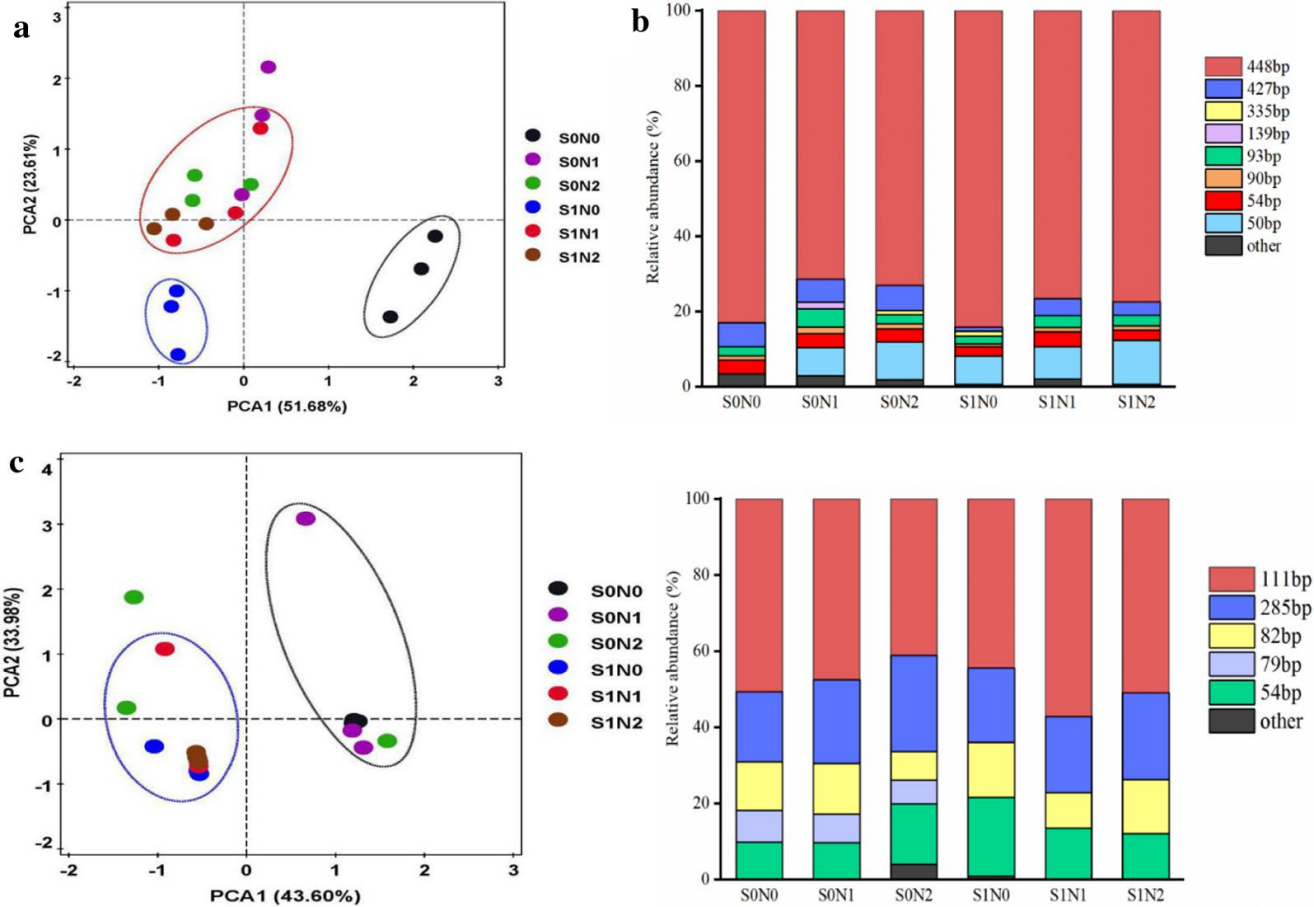
Principal Component Analysis of ammonia oxidation archaeal (**a**) and bacterial (**c**) community and the relative abundance of T-RFs for ammonia oxidation archaeal (**b**) and bacterial (**d**) in different treatments. Data were mean ± S.D. S0N0: No straw mulching with no nitrogen, S0N1: No straw mulching with 120 kg N ha^−1^, S0N2: No straw mulching with 180 kg N ha^−1^, S1N0: Straw mulching with no nitrogen, S1N1: Straw mulching with 120 kg N ha^−1^, S1N2: Straw mulching with 180 kg N ha^−1^

The Terminal restriction fragment length polymorphism (T-RFLP) analysis of *amoA* gene showed that the ammonia-oxidizing bacterial and archaeal communities changed in the different treatments. Principal component analysis (PCA) shows that the community structure of AOA was differentiated into three clusters, S0N0 as cluster 1, S1N0 as cluster 2, and other treatments as cluster 3, which indicated that straw mulching and nitrogen application resulted in variations in the AOA community (Fig. 5a). The predominant T-RFs of the AOA were 448-, 427-, and 50-bp T-RFs in all groups, which accounted for 85%, on average, of the total AOA community. Compared with the control (S0N0, S0N1, and S0N2) groups, the straw mulching (S1N0, S1N1, and S1N2) groups showed higher relative abundances of 448- and 50-bp T-RFs. By contrast, the relative abundance of 427-bp T-RF was higher in the control groups. In addition, we found some common minor *amoA* T-RFs (< 5%). For example, the 54-bp T-RF had the highest abundance in straw mulching (S1N1), while the highest relative abundances of 90- and 93-bp T-RFs were observed in S0N1 (Fig. 5b). As for the AOB community composition, PCA showed clear differences in the effects of straw mulching and nitrogen application (Fig. 5c). The T-RFs of 285-, 111-, 82-, and 54-bp were dominant in all treatments. The relative abundances of 82-, 54- and 111-bp T-RFs were higher in S1N0 and S1N1, and the highest relative abundance of 285-bp T-RF was observed in S0N2. We found that the 79-bp T-RF was only detected in the control groups, indicating that straw mulching had a great impact on the composition of AOB community (Fig. 5d).

In the in *silico* T-RFLP analysis of the *amoA* sequences obtained in this study and from the NCBI database, the ammonia oxidizers in the clone library were generally similar to those detected in the actual T-RFLP. The ammonia oxidation archaeal dominant T-RFs of 448-bp was consistent with *Thaumarchaeote*, the 54-, 93-, 139-, and 427-bp were consistent with *Crenarchaeote*. As for AOB, the dominant T-RFs of 111 bp was consistent with *Nitrosospira* sp, the 89 bp T-RF was consistent with *Nitrosomonadales*, and the T-RFs of 58- and 79-bp were consistent with *β-proteobacterium*.

### Phylogenetic analysis

**Fig. 6:**
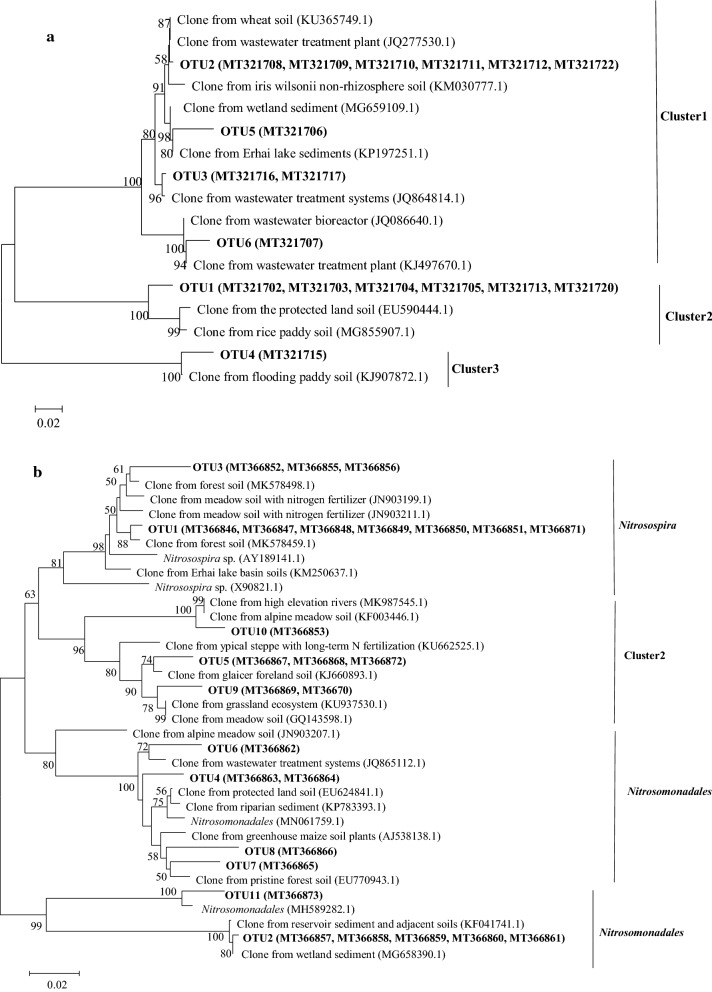
Neighbor-joining trees based on partial archaeal *amoA* genes (**a**) and bacterial *amoA* genes (**b**). Names of sequences obtained in this study are marked in bold, the numbers in parenthesizes are their respective T-RF sizes belonging to the OTU. Bootstrap analysis of 1000 replicates values are shown next to each node

In the phylogenetic analysis, using the MOTHUR Program, sequences were grouped by OTUs with a 97% similarity cut-off, resulting in 6 AOA OTUs and 11 AOB OTUs, and the AOA *amoA* gene sequences were grouped into three clusters (Fig. 6). OTU2, OTU3, OTU5 and OTU6 as cluster 1, OTU1 as cluster 2, OTU4 as cluster 3. In cluster 1, the OTUs were similar with environmental clones from sediments, and OTU2 and OTU3 were detected in all treatments, while OTU5 was uniquely found in the straw mulching with high nitrogen treatment (S1N2), and OTU6 was detected in high nitrogen treatments (S0N2 and S1N2). The OTUs that grouped into clusters 2 and 3 were similar to environmental clones from agricultural soils, and OTU1 was distributed across all the treatments, while OTU4 was uniquely found in S0N2 and S1N1 treatments (Fig. 6a; Additional file 1: Table S1). Moreover, the AOB *amoA* gene sequences were grouped into four clusters. OTU1 and OTU3 were related to *Nitrosospira* cluster, OTU5, OTU9, and OTU10 were affiliated with the cluster2, OTU2, OTU4, OTU6, OTU7, OTU8, and OTU11 were belong to the *Nitrosomonadales* cluster (Fig. 6b; Additional file 1: Table S2). Overall, the dominant AOB OTUs were affiliated with the *Nitrosospira* and *Nitrosomonadales*, and they were distributed across different treatments.

### Relationships of the ammonia oxidizers community compositions and soil properties

**Fig.7:**
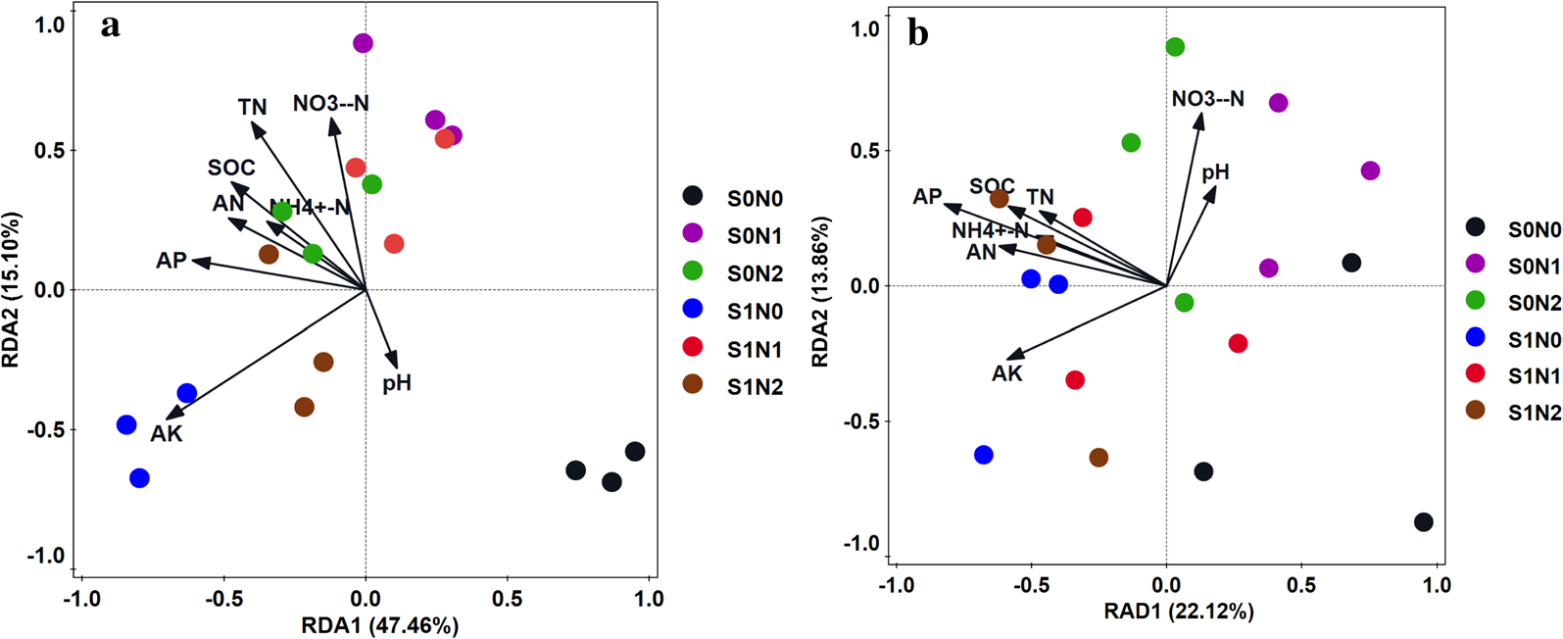
Redundancy analysis (RDA) of the correlation of soil properties with AOA community structure (**a**) and AOB community structure (**b**). S0N0: No straw mulching with no nitrogen, S0N1: No straw mulching with 120 kg N ha^−1^, S0N2: No straw mulching with 180 kg N ha^−1^, S1N0: Straw mulching with no nitrogen, S1N1: Straw mulching with 120 kg N ha^−1^, S1N2: Straw mulching with 180 kg N ha^−1^

The influences of soil properties under the different tillage regime distribution of AOA and AOB communities were evaluated using redundancy analysis (RDA; Fig. 7). Selected soil properties were used as environmental variables, and the T-RFs of AOA and AOB were used for analysing the communities of AOA and AOB. For AOA, the first and second axes accounted for 44.46% and 15.10% of the total variation between soil properties and AOA community, respectively. The distribution of AOA T-RFs was significantly correlated with SOC, TN, AN, AP, and AK, as well as NH_4_^+^-N (*P* < 0.05). Among the measured soil properties, SOC and TN were the highest contributors, accounting for 30.5% and 35.2%, respectively (Fig. 7a; Additional file 1: Table S3). These were the key factors in shaping the AOA community composition. For AOB, the first and second axes accounted for 22.12% and 13.86% of the total variation between soil properties and AOB community, respectively. SOC and AP were significantly correlated with the distribution of AOB T-RFs (*P* < 0.05), and the contribution of SOC and AP were 39.7% and 35.6%, respectively (Fig. 7b; Additional file 1: Table S4). These results suggested that SOC and AP were the major factors affecting the AOB community composition.

## Discussion

### Effects of straw mulching and nitrogen application on soil properties of alkaline purple soil

Previous study reported that purple soil had low content of SOC and AP (Xiao et al. 2016), and most of the phosphorus combined with Ca carbonate in alkaline soil, thereby reducing the availability of phosphorus (Li et al. 2011). Straw returning with nitrogen fertilizer promoted the decomposition of straw and increased the SOC (Akhtar et al. 2019), thereby significantly improving soil health (Jha et al. 2020). In present study, both straw mulching and nitrogen application significantly increased SOC content in alkaline purple soil, which was possibly owing to the slow decomposition of straw (Naresh et al. 2018), and the increase in the nitrogen fertilizer level might promote soil carbon sink, which could have resulted from the increase in aboveground biomass, especially the increase of root biomass (Rasse et al. 2005). Meanwhile, our results show that straw mulching slightly increased the content of AP. In addition, many studies have shown that tillage practices and stubble management play an important role in the soil nitrogen pool. No tillage with straw mulching increased the TN content of the topsoil and affected soil nitrogen cycle (Ye et al. 2019). In present study, straw mulching treatment resulted in a 6.60% and 4.01% increase in TN and AN content, respectively. The reason may be that the large amount of SOC released by the decomposition of straw, which increased labile organic C and N and eased the nutrient limitation of microorganisms, further increased the transformation of soil nitrogen (Dong et al. 2019). The present study found the contents of NH_4_^+^-N and NO_3_^−^-N in straw mulching groups decreased significantly during wheat harvest compared with the no straw mulching groups. Therefore, no tillage with straw mulching significantly reduced the content of mineral nitrogen in the surface soil and slowed down the deep accumulation of nitrate nitrogen in the soil to a certain extent (Ye et al. 2019; Dong et al. 2019). The present study also confirmed that straw mulching and fertilization increased the content of AK. Moreover, SOC, TN, AN, NH_4_^+^-N and AP contents were positively correlated with each other. In summary, straw mulching and nitrogen application is an effective and sustainable management practice to improve soil fertility comprehensively in alkaline purple soil.

### *amoA* gene abundance in response to years of straw mulching and fertilization

Previous studies have provided evidence that AOB and AOA functionally dominated the nitrification process in agricultural soil, and the *amoA* gene copy numbers of archaea and bacteria were highly associated with ammonia oxidation activity (Offre et al. 2009). Thus, quantification of gene abundance of AOA and AOB under the long-term field fertilization and straw mulching could reflect how N is being transformed and the pathways of potential N loss in agricultural systems (Munroe et al. 2016). Some studies had shown that the abundance of AOA and AOB shift in response to fertilization, tillage, and other environmental factors (Segal et al. 2017; Liu et al. 2018). Shen et al. (2008) found that fertilizer type might not be a factor in determining AOA abundance in alkaline soil, while long-term fertilizer treatments influenced AOA abundance in an acidic soil (He et al. 2007). The AOA abundance was found to be influenced by the addition of straw, but the AOB abundance varied little, in contrast to that of the AOA (Wessén et al. 2010). In present study, the abundance of both AOA and AOB in alkaline purple soil were increased by straw mulching. We also found that nitrogen application had significant effect on the abundance of AOB, which is consistent with previous findings (Zhou et al. 2014). Other studies indicated that the abundance of AOA was usually higher than that of AOB in alkaline sandy loam and purple soil (Shen et al. 2008; Zhou et al. 2016). However, our study results found that the abundance of AOB was higher than that of AOA after straw mulching and nitrogen application, while lower in no straw mulching with no nitrogen group, indicating that straw mulching and nitrogen application resulted in a shift in the predominant ammonia-oxidizers from AOA to AOB in alkaline purple soil, which may be related to the change of soil properties caused by straw mulching and nitrogen application.

### Ammonia oxidizer community compositions in response to years of straw mulching and fertilization

Although the AOA and AOB communities were significantly related to the agronomic management, their responses were different and varied greatly under different farming and fertilization systems (Segal et al. 2017; Liu et al. 2018). Wang et al. (2017) suggested that the application of different fertilizers had a great impact on soil AOB community compositions, while the fertilization system had a weaker impact on the composition of AOA community. However, some studies showed that AOA composition is sensitive to different fertilization regimes (He et al. 2007; Wessén et al. 2010), this might be influenced by agricultural management, climate and geographic conditions (Wang et al. 2017). In our study, the core of the AOA community in the different treatments were similar, yet more minor T-RFs were detected in the no straw mulching groups and the nitrogen application groups had their specific minor T-RFs (Fig. 5). This suggested that straw mulching could affect AOA community composition, and the minor T-RFs contributed to the community structural differences in alkaline purple soil. Presumably due to some AOA can use carbon dioxide as sole carbon source, so organic carbon sources have major influence on the AOA communities (Zhalnina et al. 2012). However, the dominant T-RFs contributed to the structural differences in the AOB community as both straw mulching and the application of nitrogen fertilizers had a great impact on soil AOB community composition in present study (Fig. 5). This suggested that the AOB was more responsive than AOA to the straw mulching and the application of nitrogen fertilizers in alkaline purple soil (Shen et al. 2008; Ai et al. 2013).

The phylogeny of the *amoA* gene has been used to study the community shifts and provide valuable additional information in many studies. For AOA, a pronounced different composition of AOA was observed in our study. Cluster 1 with the sequences retrieved from NCBI were closely related to cluster water and sediment, and cluster 2 were related to cluster soil as reported by previous studies (Francis et al. 2005). Cluster 3 and cluster 4 dominated exclusively in the application of nitrogen treatments, indicating that fertilizer supplies shifted the composition of AOA in alkaline purple soil (He et al. 2007). In addition, we found that the most dominant OTUs of AOA were affiliated with *Thaumarchaeote*. Previous studies found that the AOB members within *Nitrosospira* or *Nitrosomonadales* clusters were highly sensitive to fertilization, for example, the AOB related to *Nitrosospira* (Shen et al. 2008). Cluster 3 is highly enriched in the fertilized soil, and were most common in agricultural fields (Shen et al. 2008). However, previous studies have also reported that *Nitrosospira* are dominant in relatively low N environments (Ke et al. 2013). Our results showed that *Nitrosospira* dominated in the different treatments, which was consistent with previous research (Phillips et al. 2000).

### Relationships between community compositions, abundance of ammonia oxidizers, and soil properties

AOA and AOB communities are affected by multiple edaphic factors (de Gannes et al. 2014; Wang et al. 2017). Previous studies suggested that soil pH is an important factor affecting the community compositions of ammonia oxidizers (Zhou et al. 2016). However, the present study found that the composition of AOA significantly correlated with most measured soil properties, such as SOC, TN, AN, AP, NH_4_^+^-N, and AK. This explained how straw mulching increases SOC, thereby providing a constant source of substrate for growth and activity of archaeal ammonia oxidizers (Stopnisek et al. 2010). Although de Gannes et al. (2014) proposed that soil N characteristics of ammonium-N and organic N pools were the major factors affecting AOA communities, and ammonium-N as the substrate provided energy for ammonia oxidizers. It has been suggested that AOA has a higher affinity for ammonium/ammonia and, hence, grow widely at different ammonia concentrations (Zhalnina et al. 2012). In addition, some studies shown that soil P level was the variable which affected the communities of both AOA and AOB, and it was much more significant for the AOB under exogenous P application (Fierer et al. 2009). Our results showed that SOC and AP significantly related to the AOB communities, which might be the key factors affecting the AOB communities. In addition, our results confirmed that the soil pH did not significantly corrected with the abundance and community compositions of ammonia oxidizers, while the SOC and AP did. Moreover, the effect of soil factors on the abundance and community compositions of ammonia oxidizers was inconsistent. Such as the community compositions of AOA were significantly correlated with soil nitrogen (TN, AN, and NH_4_^+^-N) and AK, while the abundance of AOA correlated significantly AK, and the abundance of AOB correlated significantly soil nitrogen (TN, AN, and NH_4_^+^-N).

In conclusion, the present study revealed that straw mulching and nitrogen application changed the abundance and composition of ammonia oxidizers and improved the ability to provide nutrients in alkaline purple soil. SOC and AP were critical to the abundance and community compositions of AOA and AOB in alkaline purple soil. *Thaumarchaeote* and *Nitrosospira* sp were the dominant ammonia oxidizers in alkaline purple soil. This information is useful for assessing the sustainability of agricultural management strategies and soil quality.

## Supplementary Information

**Additional file 1: Table S1. **The numbers of archaral *amoA* OTUs in different straw mulching and fertilization treatments. **Table S2. **The numbers of bacterial *amoA* OTUs in different straw mulching and fertilization treatments. **Table S3. **The statistical significance of archaeal community with soil physicochemical properties by redundancy analysis (RDA) in different treatments. **Table S4. **The statistical significance of bacterial community with soil physicochemical properties by redundancy analysis (RDA) in different treatments. **Fig.S1.** The abundance of *amoA *gene copies of ammonia oxidation archaea ratio bacteria in the different treatments. Data were mean ± S.D. S1: straw with nitrogen, S0: no straw with nitrogen, N0: no nitrogen, N1: 120 kg N ha^-1^, N2: 180 kg N ha^-1^.M, and N represent the maize straw mulch and nitrogen levels, respectively.

## Data Availability

The sequenced archaeal and bacterial *amoA* gene sequences were deposited in the GenBank database with the accession numbers MT321702-MT321722 and MT366846-MT366873, respectively.
