# Supplementary material for: Straw mulching and nitrogen application altered ammonia oxidizers communities and improved soil quality in the alkaline purple soil of southwest China
Source: AMB Express. 2021 Apr 7;11:52. doi: 10.1186/s13568-021-01211-x (PMC8026789; doi:10.1186/s13568-021-01211-x)
Supplement: Supplementary file 1 — Additional file 1: Table S1. The numbers of archaral amoA OTUs in different straw mulching and fertilization treatments. Table S2. The numbers of bacterial amoA OTUs in different straw mulching and fertilization treatments. Table S3. The statistical significance of archaeal community with soil physicochemical properties by redundancy analysis (RDA) in different treatments. Table S4. The statistical significance of bacterial community with soil physicochemical properties by redundancy analysis (RDA) in different treatments. Fig.S1. The abundance of amoA gene copies of ammonia oxidation archaea ratio bacteria in the different treatments. Data were mean ± S.D. S1: straw with nitrogen, S0: no straw with nitrogen, N0: no nitrogen, N1: 120 kg N ha-1, N2: 180 kg N ha-1.M, and N represent the maize straw mulch and nitrogen levels, respectively. [file 13568_2021_1211_MOESM1_ESM.docx]

**AMB Express**

**Straw mulching and nitrogen application altered ammonia oxidizers communities and improved soil quality in the alkaline purple soil of southwest China**

Songhe Chen^1†^, Rencai Gao^1†^, Xiaoling Xiang^1†^, Hongkun Yang^1^, Hongliang Ma^1^, Ting Zheng^1^, Yun Xiao^1^, Xue Zhang^1^, Han Li^1^, Gaoqiong Fan^1^[[1]](#footnote-1)^*^, Yang Yu^2*^

*^1^* Key Laboratory of Crop Eco-Physiology & farming system in Southwest China, Ministry of Agriculture, *Sichuan Agricultural University*, Chengdu, 611130, Sichuan Province, PR China

*^2^ Soil and Fertilizer Institute, Sichuan Academy of Agricultural Sciences, Chengdu 610066, PR China*

**Additional file 1 List**

**Additional file 1: Tables**

**Additional file 1: Table S1**

**Additional file 1: Table S2**

**Additional file 1: Table S3**

**Additional file 1: Table S4**

**Additional file 1 FIGURES**

**Additional file 1: Fig.S1**

**Additional file 1: Tables**

**Additional file 1: Table S1** The numbers of archaral *amoA* OTUs in different straw mulching and fertilization treatments

| Treatments | OTU1 | OTU2 | OTU3 | OTU4 | OTU5 | OTU6 |
| --- | --- | --- | --- | --- | --- | --- |
| S0N0 | 2 | 3 | 1 | 0 | 0 | 0 |
| S0N1 | 2 | 3 | 1 | 0 | 0 | 0 |
| S0N2 | 3 | 2 | 1 | 1 | 0 | 1 |
| S1N0 | 4 | 1 | 1 | 0 | 0 | 0 |
| S1N1 | 2 | 1 | 1 | 2 | 0 | 0 |
| S1N2 | 3 | 1 | 1 | 0 | 1 | 1 |

Notes: S0N0: No straw mulching with no nitrogen, S0N1: No straw mulching with 120 kg urea N ha^-1^, S0N2: No straw mulching with 180 kg urea N ha^-1^, S1N0: Straw mulching with no nitrogen, S1N1: Straw mulching with 120 kg urea N ha^-1^, S1N2: Straw mulching with 180 kg urea N ha^-1^. SOC: Soil organic carbon, TN: Total nitrogen, AN: Available nitrogen, NH_4_^+^-N: Ammonium-N, NO_3_^-^-N: Nitrite-N, AP: Available phosphorus, AK: Available potassium.

**Additional file 1: Table S2** The numbers of bacterial *amoA* OTUs in different straw mulching and fertilization treatments

| Treatments | OTU1 | OTU2 | OTU3 | OTU4 | OTU5 | OTU6 | OTU7 | OTU8 | OTU9 | OTU10 | OTU11 |
| --- | --- | --- | --- | --- | --- | --- | --- | --- | --- | --- | --- |
| S0N0 | 1 | 1 | 1 | 2 | 0 | 0 | 1 | 0 | 0 | 0 | 0 |
| S0N1 | 1 | 2 | 1 | 1 | 3 | 0 | 0 | 1 | 1 | 0 | 0 |
| S0N2 | 2 | 1 | 1 | 1 | 1 | 0 | 0 | 0 | 0 | 0 | 0 |
| S1N0 | 2 | 1 | 1 | 0 | 0 | 2 | 0 | 0 | 0 | 0 | 0 |
| S1N1 | 2 | 2 | 1 | 0 | 0 | 1 | 0 | 0 | 0 | 0 | 1 |
| S1N2 | 1 | 1 | 1 | 0 | 0 | 1 | 0 | 0 | 0 | 1 | 0 |

**Additional file 1: Table S3** The statistical significance of archaeal community with soil physicochemical properties by redundancy analysis (RDA) in different treatments.

| Index | Contribution (%) | F | P |
| --- | --- | --- | --- |
| SOC | 30.5 | 5.1 | 0.002 |
| TN | 35.2 | 6.2 | 0.002 |
| AN | 28.6 | 4.7 | 0.006 |
| AP | 25.8 | 4.1 | 0.006 |
| NH_4_^+^-N | 23.5 | 3.7 | 0.02 |
| AK | 21.5 | 3.3 | 0.028 |
| NO_3_^-^-N | 16.7 | 2.5 | 0.052 |
| pH | 8.8 | 1.2 | 0.298 |

**Additional file 1: Table S4** The statistical significance of bacterial community with soil physicochemical properties by redundancy analysis (RDA) in different treatments

| Index | Contribution (%) | F | P |
| --- | --- | --- | --- |
| SOC | 39.7 | 3.8 | 0.006 |
| AP | 35.6 | 3.3 | 0.026 |
| AK | 25.6 | 2.2 | 0.076 |
| AN | 24.5 | 2.1 | 0.072 |
| TN | 22.5 | 1.9 | 0.124 |
| NH_4_^+^-N | 17.2 | 1.4 | 0.23 |
| NO_3_^-^-N | 16.4 | 1.4 | 0.246 |
| pH | 6.8 | 0.5 | 0.662 |

**Additional file 1: Figures**


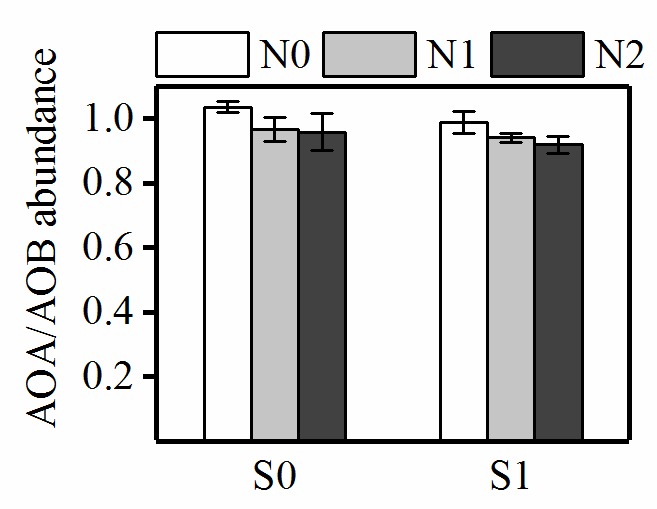


**Fig.S1** The abundance of *amoA* gene copies of ammonia oxidation archaea ratio bacteria in the different treatments. Data were mean ± S.D. S1: straw with nitrogen, S0: no straw with nitrogen, N0: no nitrogen, N1: 120 kg N ha^-1^, N2: 180 kg N ha^-1^.M, and N represent the maize straw mulch and nitrogen levels, respectively.

1. * Corresponding author: fangao20056@126.com (G. Fan). [yangyu0221@139.com](mailto:yangyu0221@139.com) (Y. Yu).

   † These authors contributed equally to this work. [↑](#footnote-ref-1)
